# A randomized two arm phase III study in patients post radical resection of liver metastases of colorectal cancer to investigate bevacizumab in combination with capecitabine plus oxaliplatin (CAPOX) vs CAPOX alone as adjuvant treatment

**DOI:** 10.1186/1471-2407-10-545

**Published:** 2010-10-11

**Authors:** Nikol Snoeren, Emile E Voest, Andre M Bergman, Otilia Dalesio, Henk M Verheul, Rob AEM Tollenaar, Joost RM van der Sijp, Sander B Schouten, Inne HM Borel Rinkes, R van Hillegersberg

**Affiliations:** 1 Department of Surgical Oncology, University Medical Center Utrecht, Heidelberglaan 100, 3584 CX Utrecht, the Netherlands; 2 Department of Medical Oncology, University Medical Center Utrecht, Heidelberglaan 100, 3584 CX Utrecht, the Netherlands; 3 Department of Medical Oncology, Antonie van Leeuwenhoek Hospital, Plesmanlaan 121, 1066 CX Amsterdam, the Netherlands; 4 Department of Biometrics, Antonie van Leeuwenhoek Hospital, Plesmanlaan 121, 1066 CX Amsterdam, The Netherlands; 5 Department of Medical Oncology, VU Medical Center De Boelelaan 1117, 1081 HV Amsterdam, the Netherlands; 6 Department of Surgery, Leiden, Albinusdreef 2, 2333 ZA Leiden, the Netherlands; 7 Department of Surgery, Lijnbaan 32, 2512 VA Den Haag, the Netherlands

## Abstract

**Background:**

About 50% of patients with colorectal cancer are destined to develop hepatic metastases. Radical resection is the most effective treatment for patients with colorectal liver metastases offering five year survival rates between 36-60%. Unfortunately only 20% of patients are resectable at time of presentation. Radiofrequency ablation is an alternative treatment option for irresectable colorectal liver metastases with reported 5 year survival rates of 18-30%. Most patients will develop local or distant recurrences after surgery, possibly due to the outgrowth of micrometastases present at the time of liver surgery. This study aims to achieve an improved disease free survival for patients after resection or resection combined with RFA of colorectal liver metastases by adding the angiogenesis inhibitor bevacizumab to an adjuvant regimen of CAPOX.

**Methods/design:**

The Hepatica study is a two-arm, multicenter, randomized, comparative efficacy and safety study. Patients are assessed no more than 8 weeks before surgery with CEA measurement and CT scanning of the chest and abdomen. Patients will be randomized after resection or resection combined with RFA to receive CAPOX and Bevacizumab or CAPOX alone. Adjuvant treatment will be initiated between 4 and 8 weeks after metastasectomy or resection in combination with RFA. In both arms patients will be assessed for recurrence/new occurrence of colorectal cancer by chest CT, abdominal CT and CEA measurement. Patients will be assessed after surgery but before randomization, thereafter every three months after surgery in the first two years and every 6 months until 5 years after surgery. In case of a confirmed recurrence/appearance of new colorectal cancer, patients can be treated with surgery or any subsequent line of chemotherapy and will be followed for survival until the end of study follow up period as well. The primary endpoint is disease free survival. Secondary endpoints are overall survival, safety and quality of life.

**Conclusion:**

The HEPATICA study is designed to demonstrate a disease free survival benefit by adding bevacizumab to an adjuvant regime of CAPOX in patients with colorectal liver metastases undergoing a radical resection or resection in combination with RFA.

**Trial Registration:**

ClinicalTrials.gov Identifier NCT00394992

## Background

Colorectal cancer (CRC) is the second leading cause of cancer-related-deaths in the western world. The incidence of CRC is still increasing [1-3]. About 50% of patients with progressed colorectal cancer develop liver metastasis [4]. The pathway from colon to liver metastases is via the portal vein and liver metastases are usually the first metastases to appear, often without signs of systemic dissemination meaning possibility of cure for these patients [5]. The median survival of patients with colorectal liver metastases is 6-12 months if untreated [6,7]. Complete surgical resection is the only treatment modality that offers hope for cure, resulting in 5 year survival for 36-60% [8-11]. Improved imaging, and surgical techniques as well as neoadjuvant therapy have increased the number of patients receiving R0 resection for colorectal liver metastasis. R0 resection is defined as a resection with tumor free margins as confirmed by the pathologist. Liver resection is a relatively safe procedure with mortality rates less that 5% [12,13]. Unfortunately only approximately 25% of patients are resectable at time of presentation. Radiofrequency ablation (RFA) is an alternative treatment option with promising five year survival rates for patients with small (< 4 cm) colorectal liver metastases. There are few studies reporting long term survival after RFA ranging from 18-30% [14-19]. The success rate of RFA greatly depends on size and open approach of the tumors treated as shown in a large meta-analysis examining 5224 treated tumors [20]. In all abovementioned studies, treated tumors had a mean diameter of less than 5 cm and patients did not have more than 3 tumors per patient on average. Surgical resection or RFA of CRLM alone is obviously not sufficient as 40%-70% of patients will develop local or distant recurrences after surgery of colorectal liver metastasis.

Different clinical studies comparing surgery and systemic adjuvant therapy with surgery and observation demonstrate a benefit in disease free survival (DFS) for the treatment arm [21-24]. Adding chemotherapy after resection might prevent the outgrowth of micrometastases present in the liver at the time of resection [25]. Portier and colleagues published the results of the first randomized controlled phase III study comparing surgery with observation with surgery and adjuvant chemotherapy with 5 FU/LV, demonstrating a disease free survival benefit for the systemic chemotherapy arm [24]. Chemotherapy regimens for advanced colorectal cancer have improved. FOLFOX and CAPOX have proven to be most effective regimens in the treatment of advanced colorectal cancer. In the CAPOX regimen infusional 5 FU is replaced with the oral derivate capecitabine [26]

After resection or RFA, regeneration of the liver takes place until the liver has reached its original volume. This takes about 6 months till a year [27-29]. Directly after liver resection, growth factors such as vascular endothelial growth factor (VEGF), hepatocellular growth factor (HGF), transforming growth factor (TGF), epidermal growth factor (EGF), macrophage inflammatory protein (MIP) en interleukine-6 (IL-6) are upregulated. Most of these growth factors that play a role in liver regeneration also are pro-angiogenic and involved with migration of tumor cells and tumor growth. Because of this production of many pro-angiogenic factors, the liver is a perfect environment for angiogenesis and tumor growth. Because small metastases (< 5 mm) are not detectable on CT scanning, there is a chance that micrometastases are present in the liver at the time of resection. Oxygen and other nutrients reach the tumor cells by diffusion, however to exceed beyond 2 mm, a tumor needs its own blood supply [30]. Pre-clinical studies demonstrate an excessive increase in the growth of intra-hepatic and extra-hepatic tumors after hepatectomy compared with sham operated mice [25,31,32]. Adding an anti-angiogenic target, such as bevacizumab, seems a logical next step in the attempt to further improve disease free survival (DFS) for patients with resected colorectal liver metastases. Bevacizumab is a monoclonal antibody against VEGF and has proven to be effective in patients with metastatic colon cancer [33].

The effect of adjuvant therapy can not yet be measured or predicted. In order to investigate the underlying mechanisms of recurrence and to predict which patient is most likely to benefit from adjuvant therapy, blood and tissue will be collected during and after therapy.

## Methods/design

### Primary objective

To demonstrate a disease free survival benefit by adding bevacizumab to an adjuvant regimen of CAPOX chemotherapy in patients with colorectal liver metastases undergoing radical resection or radical resection in combination with RFA.

The disease free survival is defined as the interval between randomization and recurrence of disease or death, whatever occurs first.

### Secondary objective

Secondary objectives are to compare survival, toxicity and quality of life. Survival is defined as the interval between randomization and death of any cause. The grade of toxicity will be assessed using the NCI-CTC criteria version 3.0. Quality of life will be studied by means of the EORTC QLC C30.

### Design

The Hepatica study is a two-arm, international, multicenter, randomized, comparative efficacy and safety study comparing adjuvant CAPOX vs CAPOX and bevacizumab in patients with resected or resected in combination with ablated colorectal liver metastases. Randomization is stratified according to clinical prognostication and treatment site.

### Stratification

Treatment assignment will be stratified, based on

(i) number (< 4 or ≥ 4) of liver metastases;

(ii) metachronous or synchronous liver metastases;

(iii) prior adjuvant chemotherapy or no prior adjuvant chemotherapy;

(iv) blood transfusion or no blood transfusion post liver surgery;

(v) treatment site;

(vi) neoadjuvant treatment with 3 cycles of CAPOX;

(vii) use of RFA in combination with resection

Criteria for RFA:

-largest liver tumor </= 4 cm

-number of liver tumors </= 3.

### Setting

Patients will be enrolled in 28 Dutch hospitals and 3 Swedish centers.

### Eligibility criteria

Inclusion Criteria: In order to be eligible for the trial, patients have to fulfil the following criteria:

1. Signed written informed consent obtained prior to any study-specific procedure;

2. Age ≥ 18 years;

3. Liver metastases radically resected (R0 resection) or liver metastases radically resected in combination with RFA

Criteria for RFA:

-largest liver tumor </= 4 cm

-number of liver tumors </= 3;

4. Study medication started ≥ 4 and ≤ 8 weeks post liver surgery;

5. Histologically confirmed liver metastasis of colorectal cancer after surgery;

6. ECOG performance status 0 or 1 (Appendix 1);

7. Adequate hematology: neutrophils ≥ 1.5 × 10^9^/L, platelets ≥100 × 10^9^/L, Hb ≥ 5.5 mmol/L, INR ≤ 1.5, APTT < 1.5 × UNL;

8. Adequate biochemistry: total bilirubin ≤1.5 UNL, ASAT and ALAT ≤ 2.5 × UNL, alkaline phosphatase ≤ 2.5 × UNL, serum creatinin ≤ 1.5 UNL.

9. Urine dipstick < 2+ for protein.

Exclusion Criteria: Patients presenting with any of the following criteria are not eligible for the study:

1 Extra-hepatic metastatic disease;

2 Adjuvant chemotherapy given < 6 months prior to detection of the liver metastases.

3 Chemotherapy for metastatic disease with the exception of max 3× CAPOX given as neoadjuvant therapy max 6 weeks before resection of the colorectal liver metastases;

4 Prior non colorectal malignancies, except adequately treated basalioma of the skin or carcinoma in situ of the cervix;

5 Bleeding diathesis or coagulation disorders or the need for full-dose anticoagulation;

6. Major surgical procedure < 4 weeks prior to start of study treatment;

7. Radiofrequency ablation without resection;

8. Females with a positive pregnancy test (within 14 days before treatment start);

9. Lactating women;

10. Fertile women (< 2 years after last menstruation) and women of childbearing potential not willing to use effective means of contraception;

11. History of psychiatric disability judged by the investigator to be clinically significant, precluding informed consent or interfering with compliance for oral drug intake;

12. Clinically significant (i.e. active) cardiovascular disease e.g. cerebrovascular accidents (≤ 6 months prior to randomization), myocardial infarction (≤ 1 year prior to randomization), uncontrolled hypertension while receiving chronic medication, unstable angina, New York Heart Association (NYHA) Grade II or greater congestive heart failure, or serious cardiac arrhythmia requiring medication;

13. Lack of physical integrity of the upper gastro-intestinal tract, malabsorption syndrome, or inability to take oral medication;

14. Known peripheral neuropathy, including oxaliplatin-induced neuropathy > grade 1. Absence of deep tendon reflexes as the sole neurological abnormality does not render the patient ineligible;

15. Organ allografts requiring immunosuppressive therapy;

16. Serious, non-healing wound, ulcer, or bone fracture;

17. Chronic treatment with corticosteroids (dose of ≥ 10 mg/day methylprednisolone equivalent excluding inhaled steroids);

18. Serious intercurrent infections (uncontrolled or requiring treatment);

19. Current or recent (within the 28 days prior to randomization) treatment with another investigational drug or participation in another investigational study;

20. Patients with known allergy to Chinese hamster Ovary cell proteins or other recombinant human or humanized antibodies or to any excipients of bevacizumab formulation, platinum compounds or to any other component of the study drugs.

### Randomization

After having properly checked all eligibility criteria, stratification parameters and having obtained patient's written informed consent, patients will be randomized by fax at the Trial Office IKO Nijmegen (fax 024-3619080). Randomized treatment will be confirmed by fax or email within one (1) working day. Randomization takes place wit TENALEA, this program uses a minimization procedure.

### Ethics

This study is to be conducted according to globally accepted standards of Good Clinical Practice, and in agreement with the latest revision of the Declaration of Helsinki (2000, including the notes of clarification 2002 and 2004) and local regulations.

This protocol has been submitted and approved by the Ethical Committee (EC) of the University Medical Centre Utrecht http://www.umcutrecht.nl/MeTC and the Central Committee on Research Involving Human Subjects http://www.ccmo.nl in accordance with Dutch legal requirements. The independent medical ethics committees of all participating hospitals have approved the study protocol

Administrative changes to the protocol are minor corrections and/or clarifications that have no impact on the study conduct. The EC may be notified of administrative changes at the discretion of the investigator. Oral and written informed consent in form is obtained from all patients prior to randomization.

### Safety

All serious adverse events during the study period, whether or not considered by the Investigator to be related to study treatment, must be reported by fax to the central data management office (Trial Office IKO, 024-361 90 80) within 24 hours using the completed SAE report. The principal investigators are responsible for the management of the safety reporting requirements according to the local regulations and guidelines. Copies of all report submissions by the principal investigators to regulatory authorities and to the ethical committee that has approved the study will be provided to the pharmacovigilance department of the license holders of the study drugs. If necessary, additional information and clarifications on cases will be forwarded to the license holders by the principal investigator.

An independent monitoring committee will discuss all reported (serious) adverse events.

### Monitoring

A data and safety monitoring board will monitor the recruitment, the reported serious adverse events and the data quality at least every 2 months. Relevant information will be included in regular study reports, and will be made available to an Independent Data Monitoring Committee (IDMC), which will be independent of the trial organizers.

The IDMC will review the data and safety data on a regular basis and report their findings to the principal investigator. The principal investigator will submit these reports to the ethics committee.

#### Data quality assurance

Data forms will be entered in the database of the NKI-AVL Data centre by a double data entry procedure. Computerized and visual consistency checks will be performed on newly entered forms; queries will be issued in case of inconsistencies.

#### On-site quality control

The sponsor will perform on-site monitoring. The monitoring visits will be scheduled at a frequency of about 1 visits per site every 6 months which may be adapted according to the site accrual.

The aim of on-site visits will be:

-To evaluate the local facilities available to the responsible investigator for performing clinical trials and to comply to all requirements of the present protocol;

-To assess the consistency of the data reported on the CRF with the source data (source data verification);

-To check that all SAE's have been properly reported;

-To resolve all previous unanswered queries.

### Interim Analysis

An interim analysis will be conducted 12 months after all participating hospitals have started the inclusion of patients or 18 months after inclusion of the first patient. An independent data monitoring committee (IDMC) will assess:

1. Safety, especially any unexpectedly high frequency of serious adverse events;

2. Data compared with previous experience which are the basis of the current the trial;

3. Any safety or efficacy data that do not justify the continuation of the trial as planned, including statistically significant differences between treatments.

The IDMC will decide on the impact of any findings for the continuation of the trial.

### Statistical Analysis

All statistical analyses will be done according to the intention-to-treat principle, i.e. all eligible patients will be included in the analysis in the arm to which they were randomized independently of whether they received the assigned treatment or not. The final analysis will be performed when 191 events (recurrences or deaths) will be observed. It is estimated that this will be possible approximately 1 year after the last patient is randomized in the study. In the final analysis tables presenting the distribution of the stratification and other important factors of all cases entered by treatment arm will be included.

Disease free survival and survival curves will be constructed by means of the Kaplan Meier technique. Curves by treatment arm will be compared using the log rank test. To adjust for possible confounding factors, the treatment effect will also be estimated by adjusting Cox's proportional hazard regression model.

Clinical and laboratory toxicity graded according NCI-CTC (version 3.0) will be collected for all patients. Comparisons by treatment of the continuous variables will be done by means of Student's t-test. Comparisons of categorical date (e.g. grades of toxicity) will be done by the chi-square test.

Quality of life will be measured using the QLC-C30 questionnaire will be completed by the patients between surgery and the start of adjuvant treatment and every 6 months thereafter, until 2 years after surgery. Quality of life assessment is discontinued when adjuvant treatment is discontinued (i.e. in case of progression in arm A or B or when off-study for any reason).

Changes over time in quality of life items will be evaluated with a repeated measurement ANOVA using a mixed effect modeling procedure (SAS Proc Mixed). This model allows retaining in the analysis patients who drop out during follow-up. F-tests are used for testing main effects of group and time and an interaction effect of group and time

#### Sample Size

The primary endpoint of the study is the DFS measured from randomization. Patients will be randomized just after R0 resection (+/- RFA). Patients receiving 3 courses of CAPOX before resection will also be included in the study. It is expected that about 1/4 of the patients will have been treated by resection and RFA. The median DFS is estimated to be 9 months for RFA treated patients and 20 months in resection treated patients. The median of the whole mixed group is estimated to be 17 months.

Based on the results in previous recent series, it is hypothesized that the addition of bevacizumab to CAPOX would result in a decrease of at least 1/3 of the hazard of progression (HR = 0.67) [34-36]. The total number of events that is necessary to provide 80% power to detect such a decrease, if truly present, with a 2 tailed logrank test at 5% significance is 191. Assuming an accrual rate of 100 patients per year, a total of 300 patients would be randomized (150 receiving Bevacizumab and 150 not) in 3 years. If all patients are followed for about 1 year further after accrual is completed, 191 events would be observed and the study will have the required power.

The total duration of the study would then be 4 years and patients would have been followed for 1 to 4 years, depending on whether they were included at beginning or by the end of the accrual in the study.

### Follow-up

#### Assessments during Study Treatment Phase

-Adverse events will be collected continuously during the Study Treatment Phase and followed until the event is either resolved or adequately explained, even after the patient has completed his/her study treatment.

-Concomitant diseases/treatment and compliance to study drugs will be monitored continuously during the Study Treatment Phase.

-History, vital signs, weight, ECOG Performance Status, urinalysis (dipstick) will be performed at each visit. Toxicity assessment, hematology, serum chemistry will be performed at each visit until cycle 8. If dipstick analysis shows ≥2+ protein, 24-hours urine needs to be collected for accurate measurement of renal protein excretion.

-ECG and chest X-ray will be performed as clinically indicated.

-Assessment for recurrence (abdominal CT, CEA measurements AND chest CT) will be done after surgery but before randomization (before cycle 1). Thereafter every 3 months in the first two years. If the patient shows signs of a recurrence/new colorectal cancer (e.g. clinical status OR rising CEA), possible re-operation or/and further cancer therapy will be recorded.

-Nature and duration of any hospitalization, treatment of any adverse event and nature and duration of any outpatient care will be recorded during the Study Treatment Phase.

#### Assessment during Follow up Phase

-CEA determinations will be done every 3 months after surgery for the first two years and thereafter every 6 months until a confirmed recurrence/new CRC and at end of follow-up after 5 years.

-Assessment for recurrence (abdominal CT AND chest CT) will be done every 3 months for the first two years after cycle 8 of chemotherapy and every 6 months thereafter until a confirmed recurrence/new CRC and at the end of follow-up after 5 years, or if the patient shows signs of a recurrence/new colorectal cancer (e.g. clinical status OR rising CEA). Possible re-operation or/and further cancer therapy will be recorded.

-Extended follow-up of hypertension, proteinuria and wound healing complication until resolution.

-Additional cancer therapy to be recorded as it occurs.

### Study medication

#### Capecitabine

Capecitabine is an oral fluoropyrimidine carbamate rationally designed to generate 5-FU preferentially in tumor tissue through exploitation of high intratumoral concentrations of thymidine phosphorylase (TP), an enzyme present at significantly increased concentrations in a wide range of tumor types, including colorectal, breast and gastric cancers, compared with normal tissue [37].

Human pharmacokinetic studies have shown that after oral administration, capecitabine is readily and almost completely absorbed through the gastro-intestinal wall, thus avoiding direct intestinal exposure to 5-FU. Capecitabine is metabolized to 5-FU via a three-step enzymatic cascade, with the final stage of this conversion mediated by TP [38].

#### Oxaliplatin

Oxaliplatin is a platinum derivative in which the platinum atom is complexed with a 1,2 diaminocyclohexane (DACH) and with an oxolate ligand. It was synthesized with the goal of trying to overcome resistance to first- and second generation platinum compounds (Sanofi-Synthelabo, 2001). The mechanism of action of oxaliplatin is similar to that of cisplatin as well as other platinum (Pt) compounds. Studies conducted to date indicated that the types and percentages of Pt-DNA adducts formed by oxaliplatin were qualitatively similar to those formed by cisplatin, but preclinical data suggested several unique attributes of the cytotoxic/antitumor activity of oxaliplatin. Oxaliplatin demonstrated a broad spectrum of in vitro cytotoxicity and in vivo antitumor activity that differed from that of either cisplatin or carboplatin. Oxaliplatin was active against several cisplatin-resistant cell lines, colon carcinoma and other solid tumors that were not responsive to cisplatin. In addition, oxaliplatin in combination with 5-FU led to synergistic antiproliferative activity in several in vivo models [39].

#### Bevacizumab

Bevacizumab is a humanized monoclonal antibody targeting vascular endothelial growth factor (VEGF or VEGF-A) which is a ligand with a central role in signaling pathways controlling tumor blood vessel development and survival [40-43]. VEGF binding to cell surface receptors stimulates the process of angiogenesis, the formation of blood vessels. These receptors, VEGF receptors 1 and 2, are found on vascular endothelial cells. VEGF binding stimulates endothelial cell proliferation, migration and survival [41,44,45].

The mode of action of bevacizumab can be summarized as follows:

-Prevents the formation of new blood vessels, thereby inhibiting the growth of existing tumors and preventing metastases from developing blood supply.

-Normalizes existing tumor blood vessels. The subsequent effects include reduction of the tortuousness of tumor blood vessels and normalization of vessel permeability. The latter effect is important because tumors usually exhibit high interstitial pressure which can prevent chemotherapeutic agents penetrating tumors and accessing tumor cells, where they exert their effects. Normalization of permeability, and therefore intratumoral pressure gradients, thus, promotes chemotherapy access [46].

-Produces blood vessel breakdown, probably through inhibition of the anti-apoptotic effects of VEGF on immature endothelial cells. VEGF also has activities beyond angiogenesis, affecting immune function via inhibition of dendritic cell maturation, formation of lymph vessels and lymphatic metastasis. All of these actions indicate that VEGF may be an important target for anticancer drug development. Bevacizumab has proven to be clinically effective in fase III studies with solid tumors.

### Treatment program

Arm A (CAPOX+Bevacizumab) consists of 8 cycles of CAPOX (either all cycles postoperatively or 3 cycles preoperatively followed by 5 cycles postoperatively).

Postoperatively patients will be treated with: bevacizumab at 7.5 mg/kg, administered as an intravenous infusion over 20-30 minutes for a maximum of 48 weeks. CAPOX will be given as follows: oxaliplatin administered as a 130 mg/m2 intravenous infusion over 2 hours (day 1, every 3 weeks) in combination with capecitabine, which will be administered orally at a dose of 1000 mg/m2 twice-daily (equivalent to a total daily dose of 2000 mg/m2), with first dose the evening of day 1 and the last dose the morning of day 15, given as intermittent treatment (3-week cycles consisting of 2 weeks of treatment followed by 1 week. without treatment). After completion of the 8 CAPOX cycles, bevacizumab will be continued as single agent (7.5 mg/kg q3w) treatment. Bevacizumab will be stopped after 48 weeks

Arm B (CAPOX) consists of 8 cycles of CAPOX (either all cycles postoperatively or 3 cycles preoperatively followed by 5 cycles postoperatively). CAPOX will be given as follows: Oxaliplatin administered as a 130 mg/m2 intravenous infusion over 2 hours (day 1, every 3 weeks) in combination with capecitabine, which will be administered orally at a dose of 1000 mg/m2 twice daily (equivalent to a total daily dose of 2000 mg/m2), with first dose the evening of day 1 and the last dose the morning of day 15, given as intermittent treatment (3-week cycles consisting of 2 weeks of treatment followed by 1 week without treatment).

## Discussion

Patients undergoing radical resection or radical resection in combination with RFA for their colorectal liver metastases can be included in this randomized controlled phase III trial where they will receive adjuvant treatment with CAPOX or CAPOX and bevacizumab. There has been much debate during the development of the study whether or not to include a surgery only arm. There were no results of randomized trials that compared surgery with surgery and adjuvant treatment for patients with resectable colorectal liver metastases until November 2006. However, worldwide patients were commonly treated with adjuvant chemotherapy even in the absence of positive studies. Portier et al. demonstrated, in a phase III randomized controlled trial a disease free survival benefit for surgery and adjuvant chemotherapy with 5 FU and Leucovorin compared to surgery alone in patients with resectable colorectal liver metastases [24]. Nordlinger and colleagues presented the results of a RCT comparing surgery with surgery and peri-operative chemotherapy [47]. With this publication, the debate has shifted towards whether or not to treat this specific patient group with peri-operative chemotherapy instead of adjuvant chemotherapy. The intention to treat analysis of Nordlinger's study unfortunately demonstrated only a trend in disease free survival benefit towards the treatment arm. From the results of this study and previous adjuvant studies it cannot be concluded that peri-operative chemotherapy is better than adjuvant chemotherapy. We have amended the protocol in December 2009 to allow 3 cycles CAPOX to be administered to bridge time to surgery. However, the steering committee of the study does feel that adjuvant treatment unless proven to be inferior of peri-operative chemotherapy in a randomized trial, is preferred. Since this amendment, we also allow patients in the study that undergo resection in combination with RFA. RFA is a frequently used in addition to resection in patients where the liver volume is insufficient to resect all tumor tissue. Since local recurrence is a frequent phenomenon after RFA in larger tumors we only allow patients with tumors smaller than 4 cm [20]. Also RFA alone or RFA for more than 3 tumors is not allowed in the study.

Administering pre-operative chemotherapy to patients with resectable colorectal liver metastases and thereby postponing a possible curative treatment seems undesirable since there are many disadvantages associated with neoadjuvant treatment. Neoadjuvant chemotherapy induces liver toxicity, such as steatosis, steatohepatitis and sinusoidal changes. Patients receiving pre-operative chemotherapy increase their chance of post-operative complications significantly (25% vs 16%) [47] Some patients undergoing neoadjuvant treatment demonstrate complete radiologic response. This only translates to complete pathologic response in 3-4% of the cases [48]. Remarkably complete pathologic response is sometimes seen in patients demonstrating incomplete radiologic response. It is therefore well established that all initial metastases should be resected. Moreover, due to chemotherapeutic alterations in the liver parenchyma, the original lesions are difficult to identify. Patients who progress during chemotherapy might result in inoperable patients due to this delay. There is also the danger of operating on benign lesions. Since there is no demonstrated benefit of peri-operative chemotherapy compared to adjuvant treatment, there is no justification to jeopardize resectability. Immediate surgery is preferred and results in direct control of the hepatic tumor without additional toxicity and should therefore not be postponed when possible. Response to preoperative chemotherapy could serve as a biological marker for good prognosis. Nonetheless patients with poor response still reach 5 year survival. Adjuvant chemotherapy can overcome many of the disadvantages mentioned above with equal benefit of increasing DFS. Neoadjuvant treatment should be preserved for irresectable patients in order to reduce tumor mass and transfer a substantial number of patients to resectable. However for resectable patients, there is no trial comparing a peri-operative regimen with adjuvant chemotherapy in terms of DFS. Until the result of such a study demonstrates a benefit for either arm it seems unjustifiable to postpone a possible curative treatment.

Because pro-angiogenic factors are upregulated during liver regeneration it is conceivable that liver regeneration will be inhibited if angiogenesis is inhibited. Clinical studies show that patients receiving bevacizumab undergoing surgery have more wound healing complications than patients not receiving bevacizumab (13% vs 3,4%) [49]. This difference might be explained by the relative long half life of bevacizumab compared to the compound (PTK/ZK) used in the animal model. Fortunately a five week interval between bevacizumab treatment and resection had shown to be sufficient to diminish the effect on wound heeling and liverregeneration [50]. Unfortunately oxaliplatin has a negative impact on the liver parenchyma demonstrating in sinusoidal obstruction syndrome in 20% of all patients [51]. Ribero et al show in a retrospective study the pathological response and dilation of sinusoids in the liver of patients who have been treated with neoadjuvant Folfox in comparison with patients who where treated with bevacizumab and FOLFOX [52]. Forty-three patients received FOLFOX and 62 patients received folfox and bevacizumab. The bevacizumab group showed a significant tumor regression in comparison with the FOLFOX group. Remarkably the incidence and severity of sinusoidal dilatation reduced in the bevacizumab. This phenomenon might be due to the fact that oxaliplatin treated sinusoid cells release MMP-2 and MMP-9 which induce the destruction of extracellular matrix and degeneration of endothelial cells. VEGFG induces the expression of MMP-9. By inhibiting VEGF, MMP-9 will also be inhibited.

## Conclusion

The HEPATICA study is designed to demonstrate that adjuvant treatment with the combination of bevacizumab and CAPOX is superior to CAPOX alone as adjuvant chemotherapy in terms of disease free survival in patients with colorectal liver metastases.

## Competing interests

The authors declare that they have no competing interests.

## Authors' contributions

NS, EV, RH drafted the manuscript

AB wrote the original protocol for the study

All authors participated in the design of the study

OD performed the statistical analysis.

All authors read and approved the final manuscript.

## Key staff at coordinating centre

Richard van Hillegersberg, MD, PhD, surgeon (principal investigator)

Emile E. Voest, MD, PhD, medical oncologist (principal investigator),

Nikol Snoeren, Msc (trial coordinator)

Sonja Verkleij/Roelien Kronemeijer (trial research nurse medical oncology)

## IDCM

Prof. R. Adam, Surgeon, Hospital Paul Brousse Paris, France

Prof. Dr. E. van Cutsem, Medical Oncologist, Katholieke Universiteit Leuven, Belgium

Prof. Dr. T. Wiggers, Surgeon, University Medical Centre Groninging, the Netherlands

Prof. Dr. D.J. Richel, Medical Oncologist, Academical Medical Centre Amsterdam

Ir. H. van Tinteren, Statistician, Antonie van Leeuwenhoek Hospital Amsterdam, the Netherlands

## Participating Centres

The principal investigators of the local hospitals are mentioned below. Investigators are of the department of Surgery (S), Oncology (O) or Gastroenterology (G).

Academic Medical Centre Amsterdam: ORC Busch (S), DJ Richel (O); Amphia Hospital Breda: A Rijken (S), OJL Loosveld (O); Atrium Medical Centre Heerlen: J Wals (O); Deventer Hospital: M Liem (S), ALT Imholz (O); Diakonessenhuis Utrecht: CI Perre (S), D ten Bokkel Huinink (O); Gelre Hospital Apeldoorn EJ Hesselink (S), JM Smit (O); Jeroen Bosch Hospital Den Bosch: K Bosscha (S), JFM Pruijt (O); Leiden University Medical Centre: R Tollenaar (S), AJ Gelderblom (O); Maastricht University Medical Centre: CHC Dejong (S) RLH Jansen (O); Maxima Medical Centre Veldhoven: R Roumen (S), G Vreugdenhil (O); Meander Medical Centre Amersfoort: B van Ooijen (S), C Rodenburg (O); Medical Centre Alkmaar: CH Smorenburg; Medical Centre The Hague: JRM van der Sijp (S), HM Oosterkamp (O); Medical Centre Leeuwarden JPEN Pierie (S), MB Polée (O); Medical Spectrum Twente Enschede: JM Klaase (S), MCJC Legdeur (O); Onze Lieve Vrouwe Gasthuis Amsterdam: PJ Borgstein (S), B de Valk (O); Saint Elisabeth Hospital Tilburg: JMGH van Riel (O); Slingeland Hospital Doetinchem: EW Muller (O); Antoni van Leeuwenhoek Hospital; F van Coevorden (S) A Cats (G); Tergooiziekenhuizen Blaricum/Hilversum HP van den Berg; University Medical Centre Groningen: RJ Porte (S) KP de Jong (S), GA Hospert (O); University Medical Centre Utrecht: R van Hillegersberg (S), EE Voest (O); VieCuri Venlo: AJ van der Wouw (O); VU Medical Centre Amsterdam: MP van den Tol (S) E Boven (O) Westfriesgasthuis Hoorn: JWD de Waard (O); Haga Hospital The Hague: JEA Portielje (O); Röpcke-Zweers Ziekenhuis - Saxenburg Groep Hardenberg: EA Runhaar (O); Gelderse Vallei Apeldoorn: C Sietses (S), E Balk (O)

## Pre-publication history

The pre-publication history for this paper can be accessed here:

http://www.biomedcentral.com/1471-2407/10/545/prepub

## References

[B1] Ponz dL Marino M Benatti P Rossi G Menigatti M Pedroni M Trend of incidence, subsite distribution and staging of colorectal neoplasms in the 15-year experience of a specialised cancer registry Ann Oncol 2004 15 940 946 10.1093/annonc/mdh224 15151952

[B2] Van CE Nordlinger B Adam R Kohne CH Pozzo C Poston G Towards a pan-European consensus on the treatment of patients with colorectal liver metastases Eur J Cancer 2006 42 14 2212 21 10.1016/j.ejca.2006.04.012 16904315

[B3] Boyle P Ferlay J Cancer incidence and mortality in Europe, 2004 Ann Oncol 2005 16 481 488 10.1093/annonc/mdi098 15718248

[B4] Borner MM Neoadjuvant chemotherapy for unresectable liver metastases of colorectal cancer--too good to be true? Ann Oncol 1999 10 623 626 10.1023/A:1008353227103 10442182

[B5] Sugarbaker PH Metastatic inefficiency: the scientific basis for resection of liver metastases from colorectal cancer J Surg Oncol Suppl 1993 3 158 160 10.1002/jso.2930530541 8503973

[B6] Jaffe BM Donegan WL Watson F Spratt JS Jr Factors influencing survival in patients with untreated hepatic metastases Surg Gynecol Obstet 1968 127 1 11 5657778

[B7] Bengmark S Hafstrom L The natural history of primary and secondary malignant tumors of the liver. I. The prognosis for patients with hepatic metastases from colonic and rectal carcinoma by laparotomy Cancer 1969 23 198 202 10.1002/1097-0142(196901)23:1<198::AID-CNCR2820230126>3.0.CO;2-J 5763253

[B8] Abdalla EK Vauthey JN Ellis LM Ellis V Pollock R Broglio KR Recurrence and outcomes following hepatic resection, radiofrequency ablation, and combined resection/ablation for colorectal liver metastases Ann Surg 2004 239 818 825 10.1097/01.sla.0000128305.90650.71 15166961 PMC1356290

[B9] Choti MA Sitzmann JV Tiburi MF Sumetchotimetha W Rangsin R Schulick RD Trends in long-term survival following liver resection for hepatic colorectal metastases Ann Surg 2002 235 759 766 10.1097/00000658-200206000-00002 12035031 PMC1422504

[B10] Pawlik TM Izzo F Cohen DS Morris JS Curley SA Combined resection and radiofrequency ablation for advanced hepatic malignancies: results in 172 patients Ann Surg Oncol 2003 10 1059 1069 10.1245/ASO.2003.03.026 14597445 PMC7101740

[B11] Rees M Tekkis PP Welsh FK O'Rourke T John TG Evaluation of long-term survival after hepatic resection for metastatic colorectal cancer: a multifactorial model of 929 patients Ann Surg 2008 247 125 135 10.1097/SLA.0b013e31815aa2c2 18156932

[B12] Scheele J tendorf-Hofmann A Resection of colorectal liver metastases Langenbecks Arch Surg 1999 384 313 327 10.1007/s004230050209 10473851

[B13] Fong Y Cohen AM Fortner JG Enker WE Turnbull AD Coit DG Liver resection for colorectal metastases J Clin Oncol 1997 15 938 946 9060531 10.1200/JCO.1997.15.3.938

[B14] Aloia TA Vauthey JN Loyer EM Ribero D Pawlik TM Wei SH Solitary colorectal liver metastasis: resection determines outcome Arch Surg 2006 141 460 466 10.1001/archsurg.141.5.460 16702517

[B15] Berber E Tsinberg M Tellioglu G Simpfendorfer CH Siperstein AE Resection versus laparoscopic radiofrequency thermal ablation of solitary colorectal liver metastasis J Gastrointest Surg 2008 12 1967 1972 10.1007/s11605-008-0622-8 18688683

[B16] Gillams AR Lees WR Radio-frequency ablation of colorectal liver metastases in 167 patients Eur Radiol 2004 14 2261 2267 10.1007/s00330-004-2416-z 15599547

[B17] Siperstein AE Berber E Ballem N Parikh RT Survival after radiofrequency ablation of colorectal liver metastases: 10-year experience Ann Surg 2007 246 559 565 10.1097/SLA.0b013e318155a7b6 17893492

[B18] Veltri A Sacchetto P Tosetti I Pagano E Fava C Gandini G Radiofrequency ablation of colorectal liver metastases: small size favorably predicts technique effectiveness and survival Cardiovasc Intervent Radiol 2008 31 948 956 10.1007/s00270-008-9362-0 18506519

[B19] Hur H Ko YT Min BS Kim KS Choi JS Sohn SK Comparative study of resection and radiofrequency ablation in the treatment of solitary colorectal liver metastases Am J Surg 2009 197 728 736 10.1016/j.amjsurg.2008.04.013 18789428

[B20] Mulier S Ni Y Jamart J Ruers T Marchal G Michel L Local recurrence after hepatic radiofrequency coagulation: multivariate meta-analysis and review of contributing factors Ann Surg 2005 242 158 171 10.1097/01.sla.0000171032.99149.fe 16041205 PMC1357720

[B21] Figueras J Torras J Valls C Llado L Ramos E Marti-Rague J Surgical resection of colorectal liver metastases in patients with expanded indications: a single-center experience with 501 patients Dis Colon Rectum 2007 50 478 488 10.1007/s10350-006-0817-6 17279302

[B22] Kemeny MM Adak S Gray B Macdonald JS Smith T Lipsitz S Combined-modality treatment for resectable metastatic colorectal carcinoma to the liver: surgical resection of hepatic metastases in combination with continuous infusion of chemotherapy--an intergroup study J Clin Oncol 2002 20 1499 1505 10.1200/JCO.20.6.1499 11896097

[B23] Langer B Bleiberg H Fluorouracil (FU) plus l-leucovorin (l-LV) versus observation after potentially curative resection of liver or lung metastases from colorectal cancer (CRC): results of the ENG (EORTC/NCIC CTG/GIVIO) randomized trial 2002 Ref Type: Generic

[B24] Portier G Elias D Bouche O Rougier P Bosset JF Saric J Multicenter randomized trial of adjuvant fluorouracil and folinic acid compared with surgery alone after resection of colorectal liver metastases: FFCD ACHBTH AURC 9002 trial J Clin Oncol 2006 24 4976 4982 10.1200/JCO.2006.06.8353 17075115

[B25] Panis Y Ribeiro J Chretien Y Nordlinger B Dormant liver metastases: an experimental study Br J Surg 1992 79 221 223 10.1002/bjs.1800790309 1555087

[B26] Van CE Hoff PM Harper P Bukowski RM Cunningham D Dufour P Oral capecitabine vs intravenous 5-fluorouracil and leucovorin: integrated efficacy data and novel analyses from two large, randomised, phase III trials Br J Cancer 2004 90 1190 1197 10.1038/sj.bjc.6601676 15026800 PMC2409640

[B27] McDermott WV Jr Ottinger LW Elective hepatic resection Am J Surg 1966 112 376 381 10.1016/0002-9610(66)90206-6 5917305

[B28] Monaco AP Hallgrimsson J McDermott WV Jr MULTIPLE ADENOMA (HAMARTOMA) OF THE LIVER TREATED BY SUBTOTAL (90 PERCENT) RESECTION: MORPHOLOGICAL AND FUNCTIONAL STUDIES OF REGENERATION Ann Surg 1964 159 513 519 14138195 10.1097/00000658-196415940-00006 PMC1408610

[B29] Bengmark S Engevik L Rosengren K Angiography of the regenerating human liver after extensive resection Surgery 1969 65 590 596 5774431

[B30] Folkman J Tumor angiogenesis: therapeutic implications N Engl J Med 1971 285 1182 1186 10.1056/NEJM197108122850711 4938153

[B31] Drixler TA Borel RI Ritchie ED van Vroonhoven TJ Gebbink MF Voest EE Continuous administration of angiostatin inhibits accelerated growth of colorectal liver metastases after partial hepatectomy Cancer Res 2000 60 1761 1765 10749151

[B32] Slooter GD Marquet RL Jeekel J Ijzermans JN Tumour growth stimulation after partial hepatectomy can be reduced by treatment with tumour necrosis factor alpha Br J Surg 1995 82 129 132 10.1002/bjs.1800820144 7881931

[B33] Hurwitz H Fehrenbacher L Novotny W Cartwright T Hainsworth J Heim W Bevacizumab plus irinotecan, fluorouracil, and leucovorin for metastatic colorectal cancer N Engl J Med 2004 350 2335 2342 10.1056/NEJMoa032691 15175435

[B34] Giantonio BJ Catalano PJ Meropol NJ O'Dwyer PJ Mitchell EP Alberts SR Bevacizumab in combination with oxaliplatin, fluorouracil, and leucovorin (FOLFOX4) for previously treated metastatic colorectal cancer: results from the Eastern Cooperative Oncology Group Study E3200 J Clin Oncol 2007 25 1539 1544 10.1200/JCO.2006.09.6305 17442997

[B35] Hurwitz HI Fehrenbacher L Hainsworth JD Heim W Berlin J Holmgren E Bevacizumab in combination with fluorouracil and leucovorin: an active regimen for first-line metastatic colorectal cancer J Clin Oncol 2005 23 3502 3508 10.1200/JCO.2005.10.017 15908660

[B36] Kabbinavar F Irl C Zurlo A Hurwitz H Bevacizumab improves the overall and progression-free survival of patients with metastatic colorectal cancer treated with 5-fluorouracil-based regimens irrespective of baseline risk Oncology 2008 75 215 223 10.1159/000163850 18852492

[B37] Miwa M Ura M Nishida M Sawada N Ishikawa T Mori K Design of a novel oral fluoropyrimidine carbamate, capecitabine, which generates 5-fluorouracil selectively in tumours by enzymes concentrated in human liver and cancer tissue Eur J Cancer 1998 34 1274 1281 10.1016/S0959-8049(98)00058-6 9849491

[B38] Reigner B Blesch K Weidekamm E Clinical pharmacokinetics of capecitabine Clin Pharmacokinet 2001 40 85 104 10.2165/00003088-200140020-00002 11286326

[B39] Raymond E Chaney SG Taamma A Cvitkovic E Oxaliplatin: a review of preclinical and clinical studies Ann Oncol 1998 9 1053 1071 10.1023/A:1008213732429 9834817

[B40] Achen MG Jeltsch M Kukk E Makinen T Vitali A Wilks AF Vascular endothelial growth factor D (VEGF-D) is a ligand for the tyrosine kinases VEGF receptor 2 (Flk1) and VEGF receptor 3 (Flt4) Proc Natl Acad Sci USA 1998 95 548 553 10.1073/pnas.95.2.548 9435229 PMC18457

[B41] Ferrara N Vascular endothelial growth factor and the regulation of angiogenesis Recent Prog Horm Res 2000 55 15 35 11036931

[B42] Ferrara N Role of vascular endothelial growth factor in regulation of physiological angiogenesis Am J Physiol Cell Physiol 2001 280 C1358 C1366 11350730 10.1152/ajpcell.2001.280.6.C1358

[B43] Shibuya M Structure and function of VEGF/VEGF-receptor system involved in angiogenesis Cell Struct Funct 2001 26 25 35 10.1247/csf.26.25 11345501

[B44] Ferrara N VEGF and the quest for tumour angiogenesis factors Nat Rev Cancer 2002 2 795 803 10.1038/nrc909 12360282

[B45] Neufeld G Kessler O Vadasz Z Gluzman-Poltorak Z The contribution of proangiogenic factors to the progression of malignant disease: role of vascular endothelial growth factor and its receptors Surg Oncol Clin N Am 2001 10 339 56 ix 11382591

[B46] Jain RK Normalizing tumor vasculature with anti-angiogenic therapy: a new paradigm for combination therapy Nat Med 2001 7 987 989 10.1038/nm0901-987 11533692

[B47] Nordlinger B Sorbye H Glimelius B Poston GJ Schlag PM Rougier P Perioperative chemotherapy with FOLFOX4 and surgery versus surgery alone for resectable liver metastases from colorectal cancer (EORTC Intergroup trial 40983): a randomised controlled trial Lancet 2008 371 1007 1016 10.1016/S0140-6736(08)60455-9 18358928 PMC2277487

[B48] Adam R Wicherts DA de Haas RJ Aloia T Levi F Paule B Complete pathologic response after preoperative chemotherapy for colorectal liver metastases: myth or reality? J Clin Oncol 2008 26 1635 1641 10.1200/JCO.2007.13.7471 18375892

[B49] Scappaticci FA Fehrenbacher L Cartwright T Hainsworth JD Heim W Berlin J Surgical wound healing complications in metastatic colorectal cancer patients treated with bevacizumab J Surg Oncol 2005 91 173 180 10.1002/jso.20301 16118771

[B50] Gruenberger B Tamandl D Schueller J Scheithauer W Zielinski C Herbst F Bevacizumab, capecitabine, and oxaliplatin as neoadjuvant therapy for patients with potentially curable metastatic colorectal cancer J Clin Oncol 2008 26 1830 1835 10.1200/JCO.2007.13.7679 18398148

[B51] Morris-Stiff G Tan YM Vauthey JN Hepatic complications following preoperative chemotherapy with oxaliplatin or irinotecan for hepatic colorectal metastases Eur J Surg Oncol 2008 34 609 614 17764887 10.1016/j.ejso.2007.07.007

[B52] Ribero D Wang H Donadon M Zorzi D Thomas MB Eng C Bevacizumab improves pathologic response and protects against hepatic injury in patients treated with oxaliplatin-based chemotherapy for colorectal liver metastases Cancer 2007 110 2761 2767 10.1002/cncr.23099 17960603

